# Early mortality in a cohort of people living with HIV in Rio de Janeiro, Brazil, 2004–2015: a persisting problem

**DOI:** 10.1186/s12879-022-07451-x

**Published:** 2022-05-17

**Authors:** Pedro H. A. C. Leite, Lara E. Coelho, Sandra W. Cardoso, Ronaldo I. Moreira, Valdilea G. Veloso, Beatriz Grinsztejn, Paula M. Luz

**Affiliations:** 1 Escola Nacional de Saúde Pública Sergio Arouca, Fundação Oswaldo Cruz, Rio de Janeiro, Brazil; 2 Instituto Nacional de Infectologia Evandro Chagas, Fundação Oswaldo Cruz, Av. Brasil 4365, Manguinhos, Rio de Janeiro, 21040-900 Brazil

**Keywords:** HIV, Acquired immunodeficiency syndrome, Survival analysis, Mortality, Risk factors, Cohort studies

## Abstract

**Background:**

Global mortality from AIDS-related diseases has been declining since 2005, resulting primarily from the widespread use and early initiation of combination antiretroviral therapy. Despite the significant improvements, high rates of early mortality, usually defined as that occurring within the 1st year of entry to care, have been observed, especially in resource-limited settings. This analysis draws upon data from an observational cohort of people with HIV (PWH) followed at a reference center for HIV/AIDS care and research in the city of Rio de Janeiro, Brazil, to identify the pattern and factors associated with early mortality.

**Methods:**

The study population includes PWH aged 18 or older followed at the National Institute of Infectious Diseases Evandro Chagas who were enrolled between 2004 and 2015. The primary outcome was early mortality, defined as deaths occurring within 1 year of inclusion in the cohort, considering two follow-up periods: 0 to 90 days (very early mortality) and 91 to 365 days (early mortality). Cox proportional hazards models were used to identify the variables associated with the hazard of very early and early mortality.

**Results:**

Overall, 3879 participants contributed with 3616.4 person-years of follow-up. Of 220 deaths, 132 happened in the first 90 days and 88 between 91 and 365 days. Very early mortality rate ratios (MRR) show no statistically significant temporal differences between the periods 2004–2006 to 2013–2015. In contrast, for early mortality, a statistically significant decreasing trend was observed: mortality rates in the periods 2004–2006 (MR = 5.5; 95% CI 3.9–7.8) and 2007–2009 (MR = 3.9; 95% CI 2.7–5.7) were approximately four and three-fold higher when compared to 2013–2015 (MR = 1.4; 95% CI 0.7–2.7). Low CD4 count and prior AIDS-defining illness were strongly associated with higher hazard ratios of death, especially when considering very early mortality.

**Conclusions:**

The present study shows an excess of mortality in the 1st year of follow-up with no changes in the mortality rates within 90 days among PWH from Rio de Janeiro. We note the significant impact of initiating treatment with immunosuppression, as evidenced by the increased risk of death among those with low CD4 cell count and with AIDS-defining illnesses.

**Supplementary Information:**

The online version contains supplementary material available at 10.1186/s12879-022-07451-x.

## Background

Brazil, a middle-income country with an existing Unified Health System, preceded many other middle and low-income countries in providing treatment and support for people living with HIV (PWH). In 1996, combination antiretroviral therapy (ART) was provided free of charge to PWH with a CD4 cell count < 200 cells/μL or an AIDS-defining illness according to CDC 1993 criteria [1]. Over the years, Brazil’s HIV treatment efforts have changed to include newer drugs and an increasing number of salvage regimens. Furthermore, ART initiation criteria for PWH changed from 200 to 350 cells/μL in 2004 [2], from 350 to 500 cells/μL in 2008 [3], and then was made available regardless of CD4 count in 2014 [4]. The impact of treatment on overall as well as AIDS-specific mortality and morbidity has been shown in local studies from Brazil [5–7] and survival benefits have been calculated [8–10] and estimated using modeling strategies [11].

Beyond the provision of HIV care and treatment, described above, the Unified Health System (SUS, Sistema Único de Saúde), implemented in Brazil in 1988, played an important role in the success of the HIV program [12]. SUS provides a universal care through a hierarchical and regionalized health service in which the primary healthcare is conceived, for most individuals, as the first point of contact with healthcare. Relating to HIV care, SUS also encompasses voluntarily Testing and Counseling services through a network of facilities as well as laboratory monitoring. In the past, HIV care had been provided mainly by the specialized centers but now are also available within the primary healthcare [13].

However, despite the significant improvements, high rates of early mortality have been observed, especially in resource-limited settings [14, 15]. Early mortality has been defined as death occurring during the 1st year after initiating treatment, where the risk of mortality is greatest since the full effect of therapy has not yet been obtained. Mortality soon after starting ART is influenced by late presentation or severe immunodeficiency, which demonstrates weakness in healthcare programs targeting PWH [16–18]. In a 2016 national study of the continuum of care outcomes, Black ethnicity/skin color, lower education, residing in a less developed region (North and Northeast), and high levels of social vulnerability were independently associated with a higher likelihood of presenting to care with advanced disease and of not using ART [19]. In another analysis on the barriers to HIV testing, fear of stigma and discrimination stood out as important reasons for not testing [20]. Estimates of life expectancy of PWH who survive the 1st year of ART are much higher than at initiation, reflecting the importance of early treatment [21, 22].

The present study aimed to identify the pattern and factors associated with early mortality in PWH from a clinical cohort followed at a reference center for HIV/AIDS care and research in the city of Rio de Janeiro, Brazil.

## Methods

We used information from the HIV Clinical Cohort followed at the STD and AIDS Clinical Research Laboratory of the National Institute of Infectious Diseases Evandro Chagas (INI/Fiocruz), in Rio de Janeiro. The HIV Clinical Cohort is an open cohort of individuals cared for at the institution for whom a database containing sociodemographic, laboratorial, clinical, outpatient, and hospital information has been routinely collected by trained abstractors. Information is updated regularly since 1998, using medical charts (electronic since 2004) of outpatient visits, hospitalizations, laboratory test results, and pharmacy records. Information on deaths is derived from medical records and the state mortality registry. Details of the methodology were previously described [23, 24].

### Study population

For this analysis, we considered adult (18 years of age or older) PWH who started treatment in our institution between January 1, 2004, and December 31, 2015. The lower limit of the period was defined based on the 2004 update of the Brazilian guidelines recommending ART initiation with a CD4 count < 350 cells/µL or an AIDS-defining illness [2]. The upper limit references the last year in which information on dates and causes of death was systematically checked with the death registry of the State of Rio de Janeiro, thus minimizing the underreporting of deaths. Almost all patients enrolled in the cohort are from Rio de Janeiro state, 99.5%, of which 97.8% live in the metropolitan area.

### Outcome definition

The primary outcome assessed was early mortality, defined as deaths occurring within 1 year from cohort enrollment. As such, the start of follow-up was defined based on the date of the first visit at our clinic when cohort enrollment was initiated, and the end of follow-up was determined by death or censoring. We considered two follow-up periods to better characterize early mortality: 0 to 90 days (very early mortality), and 91 to 365 days (early mortality). Accordingly, for any participant, study follow-up is at most 1 year of the first clinic visit. Among non-deceased individuals, censoring was defined according to the available follow-up information. Participants with follow-up information beyond 1 year from the first clinic visit were censored at 365 days. Participants with follow-up information less than 1 year from the first clinic visit were censored on the last known contact date with the clinic, given the possibility that participants may have moved to another clinic or out of state.

### Independent covariates

We considered the following variables as potentially associated with early mortality: year of first visit (2004–2006, 2007–2009, 2010–2012, 2013–2015), age, self-reported ethnicity/skin color (White, Black, Brown), years of education (≤ 5, 6–9, ≥ 10), ART naïve before first visit (yes/no), CD4 cell count at first visit (≤ 50, 51–200, 201–350, > 350 cells/µL), viral load count at first visit (< 400, 400–999, ≥ 1000 copies/mL) and presence of prior AIDS-defining illness (yes/no) [25]. CD4 cell counts and viral load measurements were assessed using a window of 180 days before and after first visit, and if more than one measurement was available, the closest value to the first visit was assigned. Prior AIDS-defining illness was based on the 1993 Centers for Disease Control and Prevention (CDC) case definition with a window of 180 days before to 30 days after first visit.

We grouped participants into the following categories based on their gender and, among men, their sexual orientation (heterosexual, homosexual or bisexual): Cis-W: cisgender women; Trans-W: transgender women (i.e. assigned male sex at birth and who identified as a woman according to information on the medical chart); MSM: gay and bisexual men who have sex with men; and MSW: men who have sex with women. In Rio de Janeiro, as in most regions of Brazil except the South, most HIV transmissions result from sexual intercourse. Accordingly, we excluded from the analyses a small number of participants who acquired HIV through other modes of HIV acquisition.

### Statistical analysis

Participants’ characteristics are described according to the year of first visit with medians (interquartile ranges) for continuous variables and absolute and relative frequency for categorical variables. We used Chi-squared tests to evaluate differences in the study population by year of first visit. Mortality rates and 95% confidence intervals (CI) were estimated, according to the year of first visit for each participant, using Poisson regression models. We evaluated the temporal trends in the very early and early mortality rates by estimating mortality rate ratios (and 95% CI) for the periods of study assuming the last period (2013–2015) as the reference category. Cox proportional hazards models were used to identify the variables associated with the hazard of early death. Covariates found to be significantly associated with the outcome (p < 0.25) in the unadjusted models were then analyzed together in a multivariate model. All variables selected from the unadjusted model were kept in the adjusted model independently of being statistically significant or not.

The results of the Cox models were interpreted as hazard ratios [26]. To overcome the problem resulting from missing data on some variables, we used bootstrapping multiple imputation methods combined with nonparametric regression models [27, 28]. To increase the statistical efficiency of the imputation process, five independent repetitions of the imputed values database were performed [29, 30].

All analyses were performed using R software [31]. Multiple imputation was done using the Hmisc library [32]. Poisson model parameters were estimated using the basic library of R, stats [31]. The parameters of Cox proportional hazards models were obtained based on the *Hmisc* and *rms* libraries [32, 33], and the risk proportionality assumption was evaluated based on the scaled Schoenfeld residuals, available in the *survival* library [34, 35].

## Results

From January 2004 to December 2015, 3991 individuals diagnosed with HIV aged 18 or older at the time of the first visit were included in the HIV Clinical Cohort of INI/FIOCRUZ. We excluded from the analysis six observations reffering to Indigenous (N = 2) and Asian descent (N = 4) participants. As participants with non-sexual routes of HIV acquisition do not constitute a homogeneous group, making it necessary to create several categories with a small number of observations, we also excluded them from the analysis (injection drug users [N = 33], vertical transmission [N = 31], work accident [N = 9], blood transfusion [N = 32] and haemophilia [N = 1]). At the end of this process the, the study population was composed of 3879 individuals whose HIV transmission occurred through sexual contact.

Table 1 presents participants’ characteristics by year of first visit. Among demographic characteristics, there was an increase in the proportion of young people (18–24 years) and a decrease in the proportion of participants aged 35–49 years old over time, reflecting the significant drop in median age from 37 (IQR 29–43) to 33 years (IQR 27–42). Over the periods, the proportion of participants of White ethnicity/skin color decreased from 61.9 to 36.5% between 2004–2006 and 2013–2015 while the proportion of participants with 10 or more years of education increased from 49.3 to 62.0%, in the same periods. The gender/sexual preference categories remained relatively stable except for the significant increase in the number of Trans-W (0.6% in 2004–2006 to 10.8% in 2013–2015).

**Table 1:** Demographic and clinical characteristics of cohort participants according to year of first visit

Variables	2004–2006	2007–2009	2010–2012	2013–2015	Total	${\chi }^{2}$
	N (%)	N (%)	N (%)	N (%)	N (%)	p-value
Age						< 0.001
Median (IQR)	37 (29.0–43.0)	35 (28.0–42.8)	34 (28.0–43.0)	33 (27.0–42.0)	35 (28.0–43.0)	< 0.001^†^
18–24	77 (9.6)	125 (11.8)	147 (13.8)	138 (14.6)	4687 (12.6)	
25–34	273 (33.9)	394 (37.1)	388 (36.3)	372 (39.5)	1427 (36.8)	
35–49	365 (45.3)	437 (41.1)	402 (37.6)	323 (34.3)	1527 (39.4)	
50 or more	91 (11.3)	106 (10.0)	132 (12.3)	109 (11.6)	438 (11.3)	
Ethnicity/skin color						< 0.0001
White	499 (61.9)	483 (45.5)	430 (40.2)	344 (36.5)	1756 (45.3)	
Black	122 (15.1)	202 (19.0)	262 (24.5)	189 (20.1)	775 (20.0)	
Brown	184 (22.8)	369 (34.7)	352 (32.9)	394 (41.8)	1299 (33.5)	
Missing	1 (0.1)	8 (0.8)	25 (2.3)	15 (1.6)	49 (1.3)	
Years of education						< 0.0001
≤ 5	213 (26.4)	294 (27.7)	330 (30.9)	227 (24.1)	1064 (27.4)	
6–9	192 (23.8)	226 (21.3)	182 (17.0)	112 (11.9)	712 (18.4)	
≥ 10	397 (49.3)	539 (50.8)	537 (50.2)	584 (62.0)	2057 (53.0)	
Missing	4 (0.5)	3 (0.3)	20 (1.9)	19 (2.0)	46 (1.2)	
Gender/sexual preference						< 0.0001
Cis-W	240 (29.8)	346 (32.6)	322 (30.1)	213 (22.6)	1121 (28.9)	
Trans-W	5 (0.6)	34 (3.2)	29 (2.7)	102 (10.8)	170 (4.4)	
MSM	293 (36.4)	362 (34.1)	384 (45.9)	364 (38.6)	1403 (36.3)	
MSW	211 (26.2)	264 (24.9)	226 (21.1)	124 (13.2)	825 (21.3)	
Missing	57 (7.1)	56 (5.3)	108 (10.1)	139 (14.8)	360 (9.3	
ART-naïve before first visit						< 0.0001
No	230 (28.5)	165 (15.5)	216 (20.2)	248 (26.3)	859 (22.1)	
Yes	576 (71.5)	897 (84.5)	853 (79.8)	694 (73.7)	3020 (77.9)	
CD4 count (cells/µL)^1^						0.31
≤ 50	97 (12.0)	144 (13.6)	153 (14.3)	120 (12.7)	514 (13.3)	
51–200	164 (20.3)	217 (20.4)	213 (19.9)	177 (18.8)	771 (19.9)	
201–350	155 (19.2)	184 (17.3)	180 (16.8)	166 (17.6)	685 (17.7)	
> 350	328 (40.7)	439 (41.3)	461 (43.1)	430 (45.6)	1658 (42.7)	
Missing	62 (7.7)	78 (7.3)	62 (5.8)	49 (5.2)	251 (6.5)	
Viral load (copies/mL)^1^						< 0.0001
< 400	112 (13.9)	173 (16.3)	222 (20.8)	209 (22.2)	716 (18.5)	
400–999	25 (3.1)	22 (2.1)	43 (4.0)	24 (2.5)	114 (2.9)	
≥ 1.000	508 (63.0)	693 (65.3)	712 (66.6)	641 (68.0)	2554 (65.8)	
Missing	161 (20.0)	174 (16.4)	92 (8.6)	68 (7.2)	495 (12.8)	
Prior AIDS-defining illness^2^						< 0.0001
No	641 (79.5)	763 (71.8)	758 (70.9)	712 (75.6)	2874 (74.1)	
Yes	165 (20.5)	299 (28.2)	311 (29.1)	230 (24.4)	1005 (25.9)	
Total	806	1062	1069	942	3879	

INI cohort, 2004–2015 (N = 3879)

*IQR* Interquartile range

^†^p-value regarding the Brown-Mood test for median difference

^1^CD4 count and viral load were measured at first visit

^2^Prior AIDS-defining illness was defined using a window of 180 days before up to 30 days after first visit

Overall, most participants (77.9%) were ART-naïve before their first visit to INI. The proportion of participants with a CD4 cell count > 350 cells/µL at first visit increased slightly from 40.7% in 2004–2006 to 45.6% in 2013–2015. The proportion of participants with a low viral load (< 400 copies/mL) increased almost 8 percentage points over time. Finally, we observed an increase in the proportion of individuals with prior AIDS-defining illnesses from 20.5% in 2004–2006 to 29.1% in 2010–2012, followed by a reduction, reaching 24.4% in 2013–2015. Overall, 23% of participants had an AIDS-defining illness at first visit, out of which, the great majority (81%) were ART-naïve. The most prevalent types of AIDS-defining illnesses were extrapulmonary tuberculosis (28%), pulmonary tuberculosis (26%), and Pneumocystis jirovecci pneumonia (24%).

Table 2 shows the number of deaths, loss to follow-up (LTFU), and person-years of follow-up, as well as the respective mortality rates and rate ratios, according to the year of first visit and follow-up period. There were 220 deaths within the 1st year after cohort inclusion, and the vast majority occurred in the first 90 days (60%). The proportion of AIDS-related deaths was higher in the first 90 days when compared to 91 to 365 days. Approximately 87% of deaths that occurred in the first 90 days were AIDS-related, while in the 91 to 365 days follow-up period they represented 66%. As the number of deaths in the first 90 days of follow-up was higher than that observed between 91 and 365 days in all years of first visit, with exception to 2004–2006, and given that the exposure time in the first follow-up period is much shorter, mortality rates in the first 90 days were much higher regardless of the year of first visit (overall 14.2 in the first 90 days versus 3.3 between 91and 365 days) (Table 2).

**Table 2:** Number of deaths, loss to follow up (LTFU), person-years, mortality rates (MR) per 100 person years and mortality rate ratios (RR) of cohort participants, according to year of first visit and follow-up period. INI cohort, 2004–2015 (N = 3879)

Follow-up period/Year of first visit	Deaths (%)	LTFU (%)	Person-years	MR (95% CI)	RR (95% CI)
0–90 days					
2004–2006	27 (3.5)	6 (0.7)	193.5	14.0 (9.6–20.3)	0.97 (0.58–1.62)
2007–2009	34 (3.2)	16 (1.5)	254.7	13.4 (9.5–18.7)	0.93 (0.57–1.51)
2010–2012	39 (3.6)	19 (1.8)	254.6	15.3 (11.2–21.0)	1.07 (0.67–1.71)
2013–2015	32 (3.4)	23 (2.4)	222.8	14.4 (10.2–20.3)	1.00
91–365 days					
2004–2006	31 (4.0)	7 (0.9)	565.8	5.5 (3.9–7.8)	3.93 (1.95–8.78)*
2007–2009	29 (2.9)	24 (2.4)	738.5	3.9 (2.7–5.7)	2.82 (1.39–6.32)*
2010–2012	19 (1.9)	22 (2.2)	740.5	2.6 (1.6–4.0)	1.84 (0.86–4.27)
2013–2015	9 (1.0)	40 (4.5)	646.0	1.4 (0.7–2.7)	1.00

^*^p-value < 0.05

**Fig. 1:**
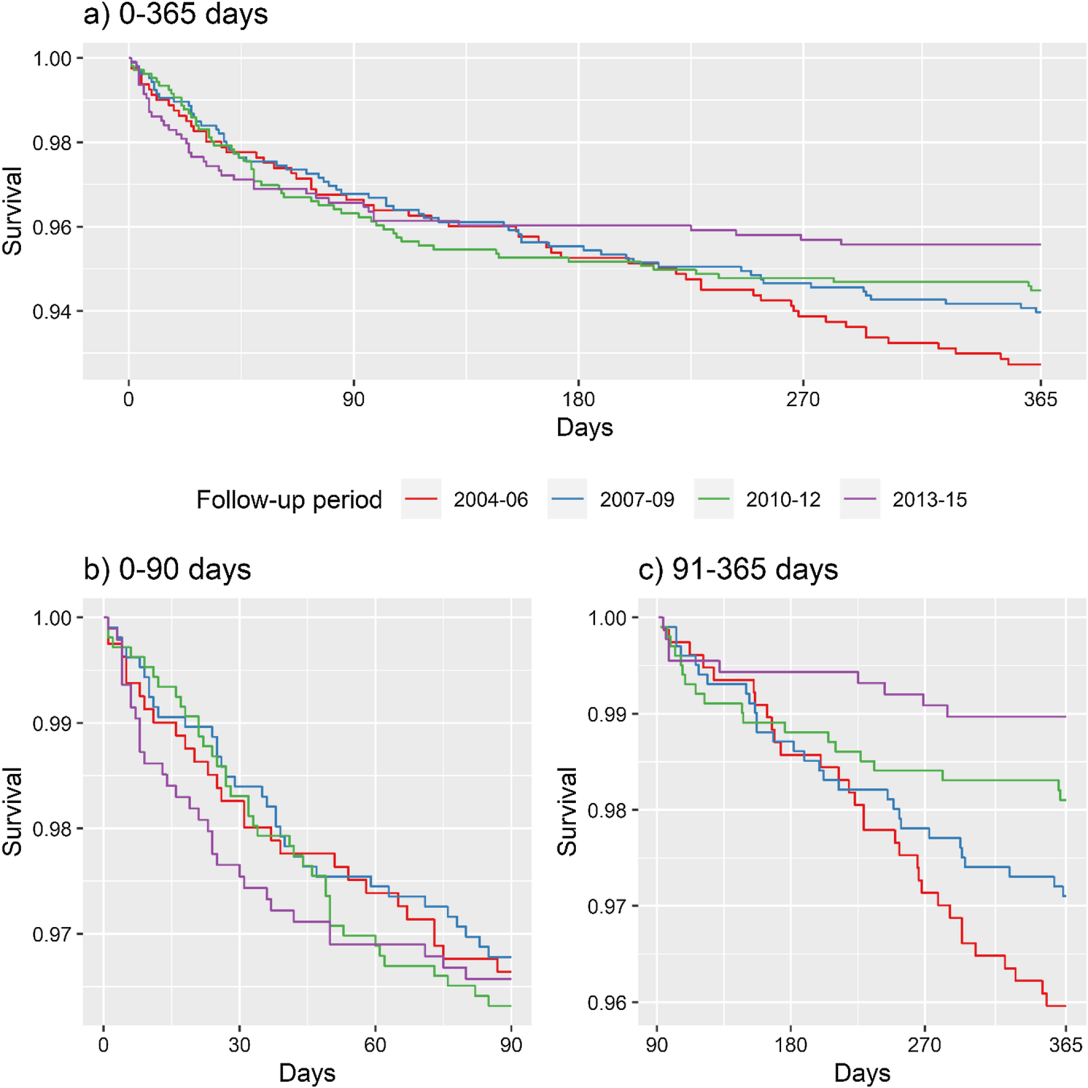
Kaplan–Meier curves, stratified by year of first visit and follow-up period of cohort participants. INI cohort, 2004–2015 (N = 3879)

Considering very early mortality rate ratios, there were no statistically significant temporal differences between the periods 2004–2006 to 2013–2015. This can also be seen at the Kaplan–Meier curves in Fig. 1. In contrast, for early mortality, a statistically significant decreasing trend was observed: mortality rates in the periods 2004–06 (RR = 3.93; 95% CI 1.95–8.78) and 2007–09 (RR = 2.82; 95% CI 1.39–6.32) were approximately three and two-fold higher than that observed in the 2013–2015 period.

Regarding LTFU, 4.0% of the participants who entered the cohort between 2004 and 2015 were LTFU, with the highest LFTU in the 2013–2015 period. This represents an LTFU rate of 4.34 per 100 person-years.

The results of Cox proportional hazards models for very early and early mortality are presented in Tables 3 and 4. The proportionality assumption was not verified for prior AIDS-defining illness in the very early mortality model nor the variables age, period of first visit, and prior AIDS-defining illness for the outcome early mortality. However, analysis of the Schoenfeld residuals plots shows no major deviations from the proportional hazards assumption (Additional file 1: Figs. S1 and S2). Besides, we can interpret the effects of these variables as an average risk ratio [36].

**Table 3:** Hazard ratios (HR) of dying in the follow-up period of 0–90 days after first visit, their respective 95% confidence intervals (CI), and p-values for Cox proportional hazard models

Variables	Unadjusted model HR (95% CI)	p-value	Adjusted model HR (95% CI)	p-value
Age				
10 year increase	1.34 (1.16–1.56)	< 0.0001	1.25 (1.05–1.47)	0.01
Ethnicity/skin color				
White	1.00	–	1.00	–
Black	1.41 (0.90–2.21)	0.14	0.97 (0.61–1.54)	0.88
Brown	1.28 (0.85–1.92)	0.24	1.06 (0.71–1.59)	0.78
Years of education				
≤ 5	2.55 (1.72–3.78)	< 0.0001	1.56 (1.02–2.39)	0.04
6–9	1.82 (1.08–3.07)	0.02	1.30 (0.77–2.21)	0.33
≥ 10	1.00	–	1.00	–
Year of first visit				
2004–2006	0.97 (0.58–1.63)	0.92	–	–
2007–2009	0.93 (0.57–1.51)	0.77	–	–
2010–2012	1.07 (0.67–1.70)	0.79	–	–
2013–2015	1.00	–	–	–
Gender/sexual preference				
Cis-W	1.00	–	1.00	–
Trans-W	0.44 (0.10–1.82)	0.25	0.84 (0.20–3.58)	0.82
MSM	1.19 (0.71–1.99)	0.50	1.43 (0.84–2.45)	0.18
MSW	1.87 (1.11–3.14)	0.02	1.14 (0.69–1.88)	0.61
ART-naïve before first visit				
No	1.00	–	1.00	–
Yes	2.89 (1.60–5.23)	< 0.001	2.52 (1.37–4.62)	< 0.01
CD4 count (cells/µL)				
≤ 50	39.92 (14.45–110.27)	< 0.0001	9.98 (3.32–29.94)	< 0.0001
51–200	22.85 (9.03–57.85)	< 0.0001	8.05 (2.98–21.77)	< 0.0001
201–350	3.05 (0.69–13.39)	0.14	1.90 (0.42–8.50)	0.40
> 350	1.00	–	–	–
Viral load				
< 400	1.00	–	1.00	–
400 a 999	1.77 (0.41–7.54)	0.33	2.47 (0.64–9.59)	0.19
≥ 1000	2.01 (1.08–3.72)	0.03	1.23 (0.65–2.33)	0.52
Prior AIDS defining illness				
No	1.00	–	1.00	–
Yes	19.27 (11.72–31.67)	< 0.0001	7.13 (4.11–12.37)	< 0.0001

INI cohort, 2004–2015 (N = 3879)

**Table 4:** Hazard ratios (HR) of dying in the follow-up period of 91–365 days after first visit, their respective 95% confidence intervals (CI), and p-values for Cox proportional hazard models

Variables	Unadjusted model HR (95% CI)	p-value	Adjusted model HR (95% CI)	p-value
Age				
10 year increase	1.70 (1.44–2.01)	< 0.0001	1.57 (1.25–1.97)	< 0.001
Ethnicity/skin color				
White	1.00	–	1.00	–
Black	1.41 (0.85–2.33)	0.18	1.64 (0.88–3.04)	0.12
Brown	0.75 (0.45–1.27)	0.29	0.93 (0.51–1.72)	0.83
Years of education				
≤ 5	2.17 (1.35–3.48)	< 0.01	1.91 (1.03–3.53)	0.04
6 a 9	1.30 (0.73–2.39)	0.40	1.04 (0.47–2.26)	0.93
≥ 10	1.00	–	1.00	–
Year of first visit				
2004–2006	3.93 (1.87–8.26)	< 0.001	4.50 (1.80–11.23)	< 0.01
2007–2009	2.82 (1.33–5.95)	< 0.01	2.59 (1.03–6.51)	0.04
2010–2012	1.84 (0.83–4.08)	0.13	1.67 (0.64–4.37)	0.30
2013–2015	1.00	–	1.00	-
Gender/sexual preference				
Cis-W	1.00	–	1.00	–
Trans-W	1.05 (0.24–4.61)	0.95	2.84 (0.63–12.82)	0.17
MSM	1.42 (0.74–2.72)	0.29	2.07 (1.01–4.24)	0.05
MSW	2.26 (1.17–4.38)	0.02	1.92 (0.96–3.82)	0.06
ART-naïve before first visit				
No	1.00		1.00	1.00
Yes	0.65 (0.42–1.03)	0.07	0.58 (0.34–0.99)	0.05
CD4 count (cells/µL)				
≤ 50	6.82 (3.62–12.85)	< 0.0001	2.45 (1.09–5.49)	0.03
51–200	2.65 (1.31–5.37)	< 0.01	1.16 (0.51–2.67)	0.72
201–350	1.54 (0.68–3.47)	0.30	0.91 (0.35–2.35)	0.85
> 350	1.00	–	1.00	–
Viral load				
< 400	1.00	–	–	–
400 a 999	1.21 (0.37–3.99)	0.76	–	–
≥ 1.000	0.82 (0.44–1.46)	0.49	–	–
Prior AIDS-defining illness				
No	1.00	–	1.00	–
Yes	5.03 (3.28–7.71)	< 0.0001	4.32 (2.33–8.00)	< 0.0001

INI cohort, 2004–2015 (N = 3747)

Variables associated with the hazard of very early mortality included all clinical variables (except for viral load), age, and education. A 10-year increase in age increased the mortality hazard by 25% (HR = 1.25; 95% CI 1.05–1.47, Table 3). Participants with 5 years or less of education had a risk of mortality 56% higher than those with 10 or more. The risk of mortality among patients who were not ART-naïve was 2.52 times that observed among those who were ART-naïve before first visit (HR = 2.52; 95% CI 1.37–4.62). Having a CD4 count lower than 50 cells/μL or between 51 and 200 cells/μL increased the hazard of mortality by approximately nine and seven-fold (HR = 9.98; 95% CI 3.32–29.94 and HR = 8.05; 95% CI 2.98–21.77, respectively) compared to those with a CD4 count above 350 cells/μL. Moreover, the hazard of mortality among participants with prior AIDS-defining illness was seven times that of participants without diagnosis (HR = 7.13; 95% CI 4.11–12.37).

All demographic variables were associated with early mortality outcome, with exception of ethnicity/skin color (Table 4). Age showed a larger effect than that observed for the very early mortality model (HR = 1.57 CI 1.25–1.97), < 5 years of education double the mortality hazard as compared to individuals with 10 years or more of education (HR = 1.91; 95% CI 1.03–3.53). MSM had a higher hazard of death when compared to cis women (HR = 2.07; 95% CI 1.01–4.24), as did MSW (HR = 1.92; 95% CI 0.96–3.82), although borderline significant. Trans-W (HR = 2.84; 95% CI 0.63–12.82) also had a higher hazard of death compared to cis-women, but not statistically significant, with a p-value of 0.17. Additionally, the period of first visit was statistically significant (reflecting the relationship found between this variable and the mortality hazard, Table 2). When considering the clinical variables, participants who had their first visit in 2004–2006 and 2007–2009 showed mortality hazard 3.5-fold (HR = 4.50; 95% CI 1.80–11.23) and 2.5-fold (HR = 2.59; CI 1.03–6.51) higher, respectively, when compared to the period 2013–2015. Participants with CD4 count ≤ 50 cells/μL had a risk of death of approximately 2.5 times (HR = 2.45; 95% CI 1.09–5.49) than of those who had a CD4 count higher than 350 cells/μL. Finally, participants with prior AIDS-defining illness at cohort entry had a three-fold increase (HR = 4.32; CI 2.33–8.00) on the hazard of mortality compared to those who did not.

## Discussion

In this study, we investigated risk factors associated with very early and early mortality in a cohort of PWH cared for at the Evandro Chagas National Institute of Infectious Diseases (INI/Fiocruz). As in previous analyses, we focused on deaths occurring within 1 year after first visit but further divided this period into two categories of follow-up: 0 to 90 days and 91 to 365 days. This analysis strategy allowed us to identify distinct trends in mortality rates per period and to assess whether different risk factors are associated with early mortality in each follow-up period. In the 12-year study period (2004–2015), there was a large expansion in the use of ART, as well as advancements in the treatment of HIV/AIDS. Globally, in this period, there was an increase in the survival of PWH though lower than that of the general population [21, 37]. This gap is even greater in the context of developing countries, where high rates of early mortality persist [14, 15, 38, 39].

Our results on the mortality rates during the 1st year highlight important excess mortality when compared with results from developed countries [17, 40, 41], but are on the same level as those reported in other developing countries [17, 42]. Regarding very early mortality, meta-analysis estimates show that the estimated mortality rate of 11.5% is much higher than the estimated for low- and middle-income countries, 6.0% [38]. However, attention should be drawn to the great heterogeneity among the populations analyzed, as mortality rates range from 1% (0.3–2.5%) in a Chinese cohort of patients recruited in 2003–2004 and followed every 6 months until May 2010 [43] to 18.4% (14.5–23.0%) in a cohort of patients in a rural hospital in Tanzania who started ART between October 2003 and November 2006 [44].

Importantly, our results show that over time very early mortality rates have remained high while there was a decreasing trend for mortality rates during 91 to 365 days of follow-up, with the periods 2010–2012 and 2013–2015 having similar mortality rates. The high very early mortality rate in all periods (between 10 to 12 per 100 person-years) may be a result of changes in the composition of the study cohort. In the last decade, HIV infection has increasingly affected people of lower socioeconomic status [45]. Among our study participants, we observed a decrease in the proportion of White participants and an increase in the proportion of Black and Brown participants. There is an accumulation of evidence about the social and economic disadvantages of the Black and Brown population compared to the White population in Brazil [46–48]. Notably, during the past decade, studies have shown an increase in the affirmation of Black and Brown identity in Brazil, with a consequent increase in the self-report as Black or Brown in National studies [49]. Nevertheless, this fact could not explain the changes observed in the composition of the cohort as they are much more pronounced than those observed in the national study cited previously. The excess of very early mortality over time may also result from health system failures in identifying PWH at an early stage of the disease [50]. Despite the advances made in terms of HIV treatment, efforts still need to be made to expand HIV surveillance system.

Regarding the survival analysis, our findings corroborate those of other studies, although some variables were evaluated differently. This includes the gender/sexual preference variable, in which MSM and MSW were at a higher risk of dying when compared to the category of ciswomen. Studies have shown that women are more likely to look for health services, which has been suggested as the main explanation for the lower mortality rates found among them in the general population [51], a fact also evidenced when it comes to early mortality among PWH [52–54]. Sexual and reproductive health needs may also play a role. Prenatal and childbirth coverage in Brazil is close to 100% and the coverage of testing for HIV infection during pregnancy is over 80% [55–57]. Men tend to be diagnosed later than women with AIDS-related illnesses, thus increasing the risk of mortality, particularly early mortality [10, 58–60]. Here, it is important to highlight the significant burden of tuberculosis in our setting, as PWH with TB tend to seek care later than expected, with low CD4 count, greatly contributing to early mortality [61, 62]. Our results have equally evidenced the burden of TB, with extrapulmonary and pulmonary TB being the two most frequent AIDS-related illnesses.

It is worth mentioning the changes in the effects of the variable gender/sexual preference after adjustment. While in the unadjusted model the MSW category is statistically significant (HR = 2.26; 95% CI 1.17–4.38) and has a higher hazard ratio than MSM (HR = 1.42; 95% CI 0.74–2.72), in the adjusted model we can observe that MSM is the category statistically significant and with higher HR (HR = 2.07; 95% CI 1.01–4.24) when compared to MSW (HR = 1.92; 95% CI 0.96–3.82). These findings are similar to those presented by Coelho and colleagues [58] while analyzing competing risks in this same cohort with participants enrolled between 2000 and 2011. Transwomen also had a substantial difference between models, with a much higher hazard ratio (HR = 2.84; 95% CI 0.63–12.82), although not statistically significant since the number of transwomen in the cohort is small (N = 170). Albeit the 2.8-fold higher hazard of mortality when compared to ciswomen, transwomen have a higher CD4 count at first visit and entered more recently in the cohort, two factors that are protective. Therefore, this can possibly be explained by non-AIDS mortality [63].

Even though the percentage of participants with CD4 count > 350 cells/µL at first visit increased from 2004–2006 to 2013–2015, it consistently remained below 50% among the periods analyzed. This is a global trend, even in high-income countries [64]. CD4 count has a substantial impact on early mortality, and, even in recent periods with better treatments and earlier initiation, deaths occurring very early after reaching care were not affected. The results obtained for the clinical indicators at first visit are consistent with other studies, revealing that participants with low CD4 cell counts and ART naïve present higher risks of early mortality [65–67]. Indeed, we show that low CD4 counts dramatically increased the hazard of mortality in the first 90 days of follow-up. In this regard, though the Brazilian recommendations for ART initiation have been for all PWH irrespective of CD4 cell count, those with low CD4 cell counts should be prioritized since they may need to initiate, in addition to ART, prophylaxis or treatment for opportunistic infections to reduce their high mortality [4].

This study has strengths and limitations that should be acknowledged. A strength is the thoroughness with which mortality information was sought after, including medical chart review and record linkage of mortality information using the State of Rio de Janeiro mortality database. Brazil instituted national reporting of mortality in 1977 and has a very robust system in place with high completeness of data regarding the cause of death, particularly in Rio de Janeiro [65]. Additionally, our institute provides primary, secondary, and tertiary care such that most deaths occurred within our institute and were thus captured locally. Nevertheless, it is important to highlight a limitation of our population with regards to how representative they are of PWH cared for at other clinics. INI is the largest HIV care provider in Rio de Janeiro state and the level and quality of care equal that of university hospitals and/or other research-based HIV services. Specifically, INI provides specialty and tertiary care, services not provided at the many primary care facilities. Given these peculiarities, PWH cared for at INI are not representative of PWH overall but can be taken as representative of those cared for at reference services of Brazil. Also noteworthy is the use of the multiple imputation techniques, thus avoiding the exclusion of participants with missing values and increasing the power of the statistical tests used in the models. Limitations include the maintenance of variables with evidence that the Cox proportional hazards assumption had not been verified [34, 68]. However, since there are limitations to proportional hazards hypothesis testing, we interpreted the effects as an average of risk ratios over the follow-up period [36, 69, 70].

## Conclusion

Based on information from a cohort of PWH followed at a reference institution in Rio de Janeiro, early mortality rates were much higher than those observed in developed countries. This excess mortality is strongly associated with advanced disease and, therefore, with the patients’ level of immunodeficiency at the time of first visit. Late presentation of PWH to care is, therefore, one of the most significant problems for care providers and PWH. Among developing countries, Brazil was the first country to make ART available to all PWH. Thus, it is of fundamental importance that public policies are designed to promote early diagnosis and linkage to care, so patients can benefit from early ART initiation, preventing the evolution of the disease, thus increasing survival and improving the quality of life of PWH.

## Supplementary Information

**Additional file 1: Figure S1.** Plots of the Schoenfeld residuals against transformed time for the very early mortality model (0 to 90 days). **Figure S2.** Plots of the Schoenfeld residuals against transformed time for the early mortality model (91 to 365 days).

## Data Availability

The datasets generated and/or analyzed during the current study are not publicly available due Institutional Review Board (IRB) restrictions but are available from the corresponding author on reasonable request.
