# Supplementary material for: Early mortality in a cohort of people living with HIV in Rio de Janeiro, Brazil, 2004–2015: a persisting problem
Source: BMC Infect Dis. 2022 May 17;22:475. doi: 10.1186/s12879-022-07451-x (PMC9115995; doi:10.1186/s12879-022-07451-x)

**Early mortality in a cohort of people living with HIV in Rio de Janeiro, Brazil, 2004-2015: a persisting problem**

Pedro H.A.C. Leite^1,2§^, Lara E. Coelho^2^, Sandra W. Cardoso^2^, Ronaldo I. Moreira^2^, Valdilea G. Veloso^2^, Beatriz Grinsztejn^2^, Paula M. Luz ^1,2^

^1^ Escola Nacional de Saúde Pública Sergio Arouca, Fundação Oswaldo Cruz, Rio de Janeiro, Brazil

^2^ Instituto Nacional de Infectologia Evandro Chagas, Fundação Oswaldo Cruz, Rio de Janeiro, Brazil

^§^ Corresponding author: Pedro Henrique Amparo da Costa Leite

Instituto Nacional de Infectologia Evandro Chagas, Fundação Oswaldo Cruz

Av. Brasil 4365, Manguinhos

Rio de Janeiro, 21040-900, Brazil

Tel +55(21)38652191

Email: pedroamparo@gmail.com

Figure S1. Plots of the Schoenfeld residuals against transformed time for the very early mortality model (0 to 90 days)


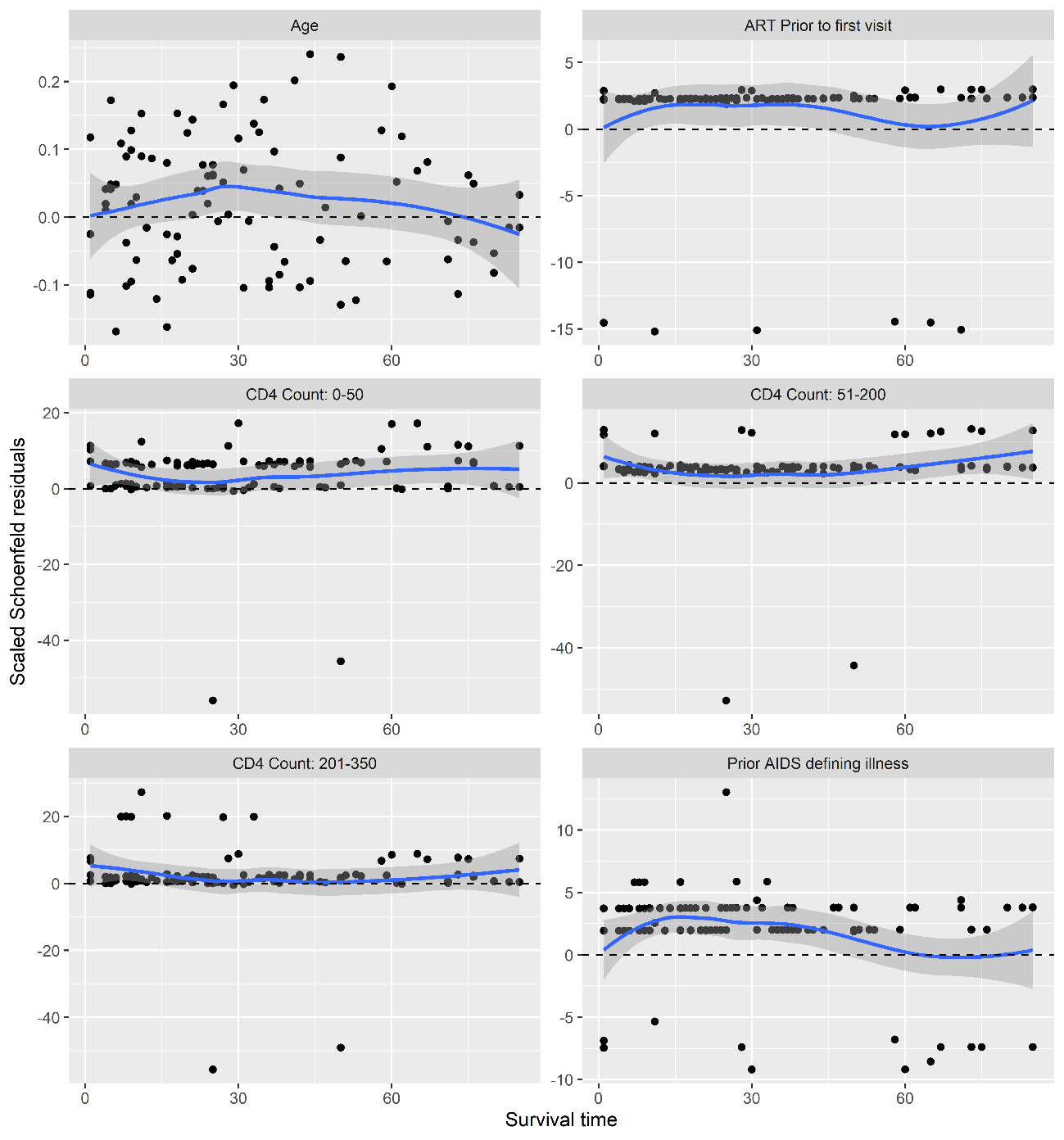


Figure S2 - Plots of the Schoenfeld residuals against transformed time for the early mortality model (91 to 365 days)


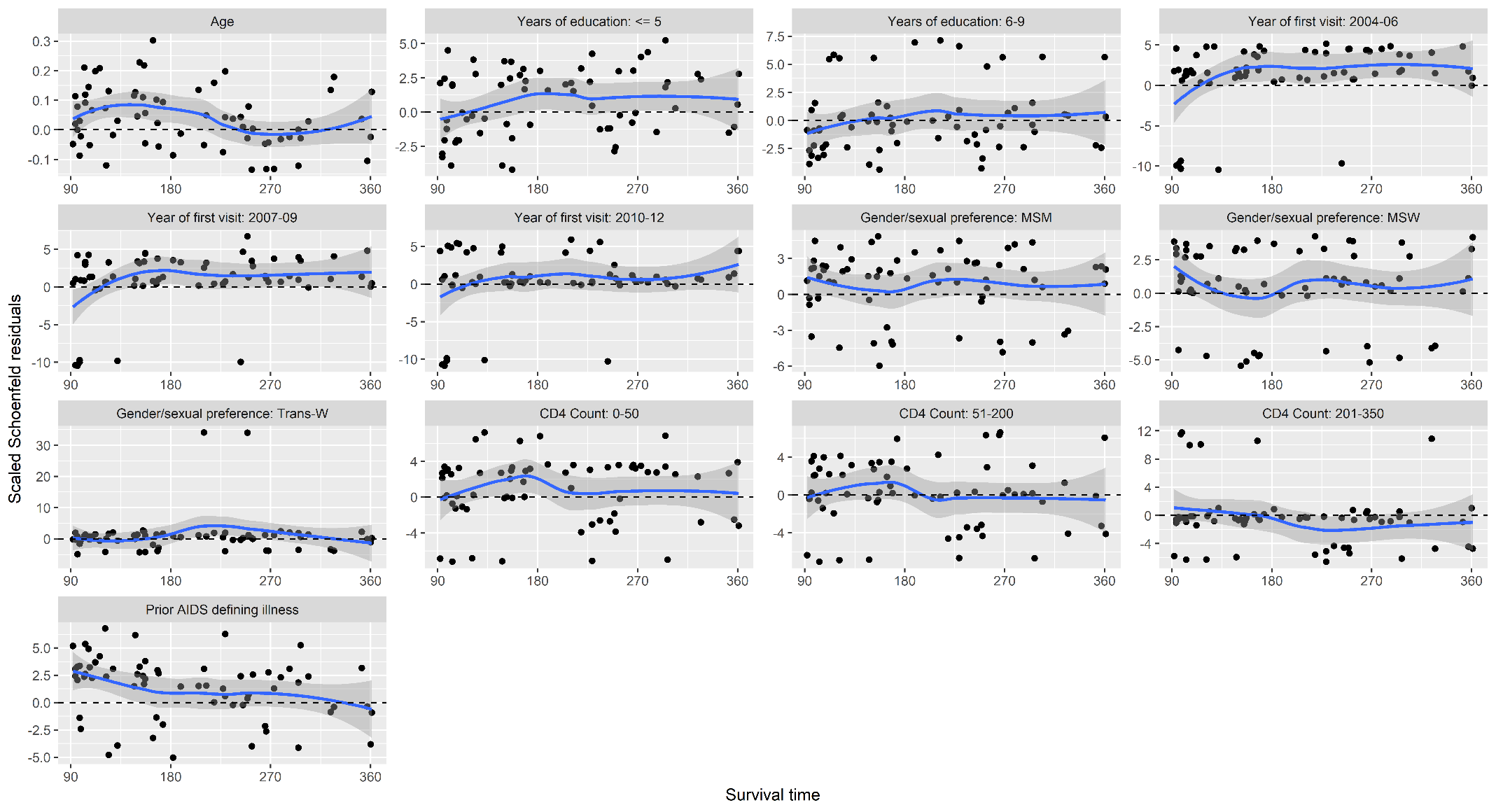

Supplement: Supplementary file 1 — Additional file 1: Figure S1. Plots of the Schoenfeld residuals against transformed time for the very early mortality model (0 to 90 days). Figure S2. Plots of the Schoenfeld residuals against transformed time for the early mortality model (91 to 365 days). [file 12879_2022_7451_MOESM1_ESM.docx]
